# Dietary High Levels of Coconut Oil Replacing Fish Oil Did Not Affect Growth, but Promoted Liver Lipid Deposition of Orange-Spotted Groupers (*Epinephelus coioides*)

**DOI:** 10.3390/ani14111534

**Published:** 2024-05-22

**Authors:** Kun Wang, Tao Song, Liner Ke, Yunzhang Sun, Jidan Ye

**Affiliations:** Xiamen Key Laboratory for Feed Quality Testing and Safety Evaluation, Fisheries College, Jimei University, Xiamen 361021, China; wangkun@jmu.edu.cn (K.W.); 202011908003@jmu.edu.cn (T.S.); 202011908019@jmu.edu.cn (L.K.); jmusunyunzhang@163.com (Y.S.)

**Keywords:** *Epinephelus coioides*, coconut oil, growth performance, fatty acid metabolism

## Abstract

**Simple Summary:**

The most prominent feature of coconut oil (CO) is that it contains over 80% saturated fatty acids and about 60% medium chain fatty acids of total fatty acids. With these characteristics, CO shows many health benefits, but concerns about the risk of cardiovascular disease still remain in dispute. So far, there has been limited research on the potential use of CO in aquafeeds, being used as an alternative to fish oil (FO), and inconsistent results were presented. Therefore, we performed an experiment to investigate the effects of CO replacing FO on the growth performance of the orange-spotted grouper (*Epinephelus coioides*) in an attempt to determine its potential use and pertinent lipid metabolic mechanism in the fish species. Results show that CO could completely replace FO without affecting the growth performance, but high CO feeding will compromise the liver lipid deposit and long chain polyunsaturated fatty acids reduction of fish flesh in orange-spotted groupers.

**Abstract:**

In this study, we conducted an 8-week feeding trial to investigate the effects of replacing fish oil (FO) with coconut oil (CO) on the growth performance, blood components, tissue fatty acid (FA) profile, and mRNA levels of genes related to lipid metabolism in the liver of the orange-spotted grouper (*Epinephelus coioides*). Five isolipidic and isoproteic diets were formulated through increasing the CO levels (0, 25%, 50%, 75%, and 100%, respectively). Triplicate groups of twenty-five fish (initial wet weight of about 22.4 g/fish) were fed one of the diets twice daily to apparent satiety. The 25% CO diet had the highest growth rate and feed utilization, and the 100% CO diet exhibited a comparable growth and feed utilization with that of the control diet, indicating a suitable FO substitute. Moreover, the hepatosomatic index, intraperitoneal fat rate, liver lipid content, as well as the serum HDL-C content and ALT activity had positive linear and/or quadratic responses, but the serum TC and LDL-C contents exhibited the opposite trend, with an increasing CO inclusion level. The FA profile in the liver and muscle generally mirrored the FA profile in the feed. Furthermore, the mRNA levels of the *fas*, *acc*, *g6pd*, *srebp-1c*, and *δ6fad* genes in the liver had positive linear and/or quadratic responses, but the mRNA levels of *elovl 4* and *elovl 5* had the opposite trend, with increasing dietary CO inclusion levels. When compared with the control diet, 25% and 50% CO diets up-regulated the mRNA levels of *cpt 1*, while the 75% and 100% CO diets down-regulated its mRNA levels. The *hsl* and *atgl* were down-regulated through the addition of dietary CO. The mRNA level of *lpl* was not affected by dietary treatments. Results showed that CO could completely replace FO without affecting growth performance, but high CO will lead to the significant liver lipid deposition and lower LC-PUFAs contents of fish flesh.

## 1. Introduction

The biggest difference between fish oil (FO) and edible vegetable oil (VO) is that the former is rich in n-3 long-chain polyunsaturated fatty acids (n-3 LC-PUFA), such as docosahexaenoic acid (DHA) and eicosapentaenoic acid (EPA), which can provide essential EFA for marine aquaculture fish [1]. Fish oil also contains some flavor components that can improve the palatability of feed and the ingestion of fish [2]. Therefore, FO has become the preferred oil ingredient for feed formulations of marine aquaculture fish [3]. However, the fast development of the global aquaculture industry, represented by China, has contributed to a sharp increase in FO consumption. This leads to a constant shortage in FO supplies, which in turn has kept prices high, thereby increasing the cost of aquatic feed [4]. Therefore, finding candidates to replace FO has become one of the major issues in the aquafeed sector. Edible VOs are widely used as a potential alternative to FO in aquafeeds due to their wide sources, huge production, and relatively low prices. So far, the application of VOs in commercial feed formulations for fish farming has made great progress [5,6,7,8,9,10]. Several results show that the effects of replacing FO with edible VOs differ due to the different fatty acid (FA) compositions of different VOs and the synergistic effects of FAs through the blending of FO and VOs [11,12,13,14,15].

Coconut oil (CO) is a VO extracted from coconut meat. It is mainly produced in Southeast Asian and South Asian countries such as Philippines, Indonesia, and India, and South American countries such as Brazil and Colombia [16]. Unlike other VOs, CO is rich in saturated fatty acids (SFAs), accounting for about 80–90% of total FAs, which makes CO a firm texture at room temperatures, which is less prone to oxidation and rancidity [17]. Medium chain fatty acids (MCFAs) are the predominant FA type of CO, mainly including lauric acid, decanoic acid, and octanoic acid, accounting for about 60% of the total FAs, with the lauric acid content being as high as 50–53% [18]. MCFAs can be quickly absorbed after digestion and immediately used as energy in the liver [19,20]. With the abundant presence of MCFAs, CO exhibits many health benefits, mainly including rapid energy supply, lipid-lowering, antiviral, antibacterial, and antioxidant properties [20,21,22,23,24,25]. Although there is a concern about the risk of cardiovascular disease caused by SFAs [26], any direct significant relationship between mortalities of cardiovascular disease and intakes of total fats, SFAs, MUFAs, and PUFAs has not yet been confirmed and is highly controversial [27,28,29]. In fish, there is limited research on the potential use of CO in aquafeeds as an alternative to FO. However, these limited studies have yielded inconsistent results. Some studies reported that CO can completely replace FO without affecting the growth of fish [11,30,31,32,33] and that a synergistic effect of a CO and FO blend on promoting growth was also observed [11,12], while others have reported that excessive CO substitution for FO can have adverse effects on fish growth [34,35,36,37,38]. This may be due to differences in fish species, feed composition, and dietary CO inclusion levels. Therefore, it is necessary to further study the role of CO in growth promotion and lipid metabolism when CO is used as a FO alternative in aquafeeds in an attempt to promote the development of sustainable aquaculture.

The grouper, an important marine carnivorous fish species, is favored by people in Southeast Asian coastal countries including China due to its high market price and benefits for intensive farming [39]. China has boosted its overall development, with an aquaculture output of 205,800 tons in 2022 [40]. There was still limited research on the potential use of VOs as an alternative to FO in feed for the fish species, and their influences on lipid metabolism were reported by our research team [10,14] and previous studies [38,41,42]. These results indicate that FO could be replaced partially or completely by VOs without affecting growth. Furthermore, feeding a 50% palm oil (PO) substitution diet resulted in maximum growth, possibly due to the synergistic effect of FO and PO when mixed in an appropriate proportion [14]. We also assume that CO and FO mixing in an appropriate proportion may have a synergistic effect, as CO is rich in well-known MCFAs and other SFAs. The orange-spotted grouper (*Epinephelus coioides*) is one of the major species of farmed grouper. In view of this, we conducted an experiment to investigate the effects of replacing dietary FO with CO on growth performance, plasma biochemical components, tissue FA composition, and the expression levels of genes related to lipid metabolism in orange-spotted grouper, attempting to determine whether dietary CO affects the growth and feed utilization and whether it directly activated the gene expressions associated with lipid metabolism in the fish species.

## 2. Materials and Methods

### 2.1. Test Feed

A basal diet was formulated to contain 50% crude protein and 12% crude lipids using a defatted fish meal, wheat gluten, soybean meal, shrimp meal, gelatin, and casein as the protein sources, and FO, CO, and soy lecithin as the main lipid sources (Table 1). The FO was replaced by CO at 0%, 25%, 50%, 75%, and 100% increments in the basal diets to prepare 5 experimental diets (0% CO, 25% CO, 50% CO, 75% CO, and 100% CO, respectively). The five experimental diets were made according to the feed preparation procedure of our previous study [14]. The pellets were dried in a ventilated oven at 65 °C for 24 h until the moisture was reduced to below 10%, and were then sealed in plastic bags and stored at −20 °C for the subsequent growth trial. The FA profiles of the test diets are presented in Table 2.

### 2.2. Feeding Management

The feeding trial was carried out at Fujian Dabeinong Fisheries Technology Company (Zhaoan County, Zhangzhou City, China). The orange-spotted grouper juveniles were maintained in a concrete pond and fed with the basal diet for 2-week acclimatization prior to the start of the trial. Groupers initially weighing 22.35 g/fish (n = 25 fish) were randomly allotted into five groups, each with triplicate tanks (500 L/tank), at a density of twenty-five fish per tank within a circulating aquaculture system equipped with a temperature control device. Groups of fish were hand-fed one of the five diets to apparent satiation twice daily (7:00 and 17:00) under a natural photoperiod across an eight-week feeding period. Uneaten feed was collected and then feces were removed via siphoning 30 min after each meal. The collected feed was then dried at 65 °C and weighed to calculate the amount of feed intake. Dissolved oxygen and water temperature were measured daily at 15:00 h and nitrite—N was monitored twice a week using a multi-parameter photome (HI83200, Hanna Instruments, Woonsocket, Rhode Island). The water temperature was kept at 29 ± 0.5 °C, the dissolved oxygen content ranged from 6.93 ± 0.65 mg/L, and ammonia nitrogen was below 0.21 mg/L throughout the feeding period.

### 2.3. Sample Collection

At the end of the experiment, fish in each tank were captured and anesthetized with 100 mg/L of MS-222 (tricaine methane sulfonate, Sigma-Aldrich Shanghai Trading Co., Ltd., Shanghai, China), then batch-weighed and counted to determine the percentages of weight gain (WG), the specific growth rate (SGR), the daily feed intake (DFI), the feed conversation rate (FCR), and survival. Fifteen fish per treatment (five fish per tank) were randomly captured and anesthetized with MS 222 (100 mg/L) and weighted individually, followed by a blood draw using a 2 mL syringe through the caudal vein. Blood samples were placed at 4 °C for 12 h before serum separation via centrifugation at 1027× *g* at 4 °C for 10 min. Serum samples were pooled by tank, and then stored in 1.5 mL Eppendorf tubes at −80 °C for the subsequent analysis of biochemical parameters. After completing the blood draw, the same batch of five fish per tank were then dissected to aseptically remove the liver and viscera in order to calculate the hepatosomatic index (HSI) and condition factor (CF), followed by the excision of dorsal muscles. For liver histological observations, 1 mm^3^ of liver (one fish per tank) was cut and fixed in 4% polyformaldehyde solution. Liver and dorsal muscles were then, respectively, pooled by tank and stored at −80 °C for the analysis of lipids, fatty acids, and gene expression. Another four fish in each tank were randomly caught and pooled in plastic bags and stored at −20 °C for the determination of the proximate composition.

### 2.4. Proximate Composition Analysis

The proximate composition of ingredients, diets, fish samples, liver, and samples were determined according to the method of the Association of Official Analytical Chemists [43]. Dry matter was measured via drying in an oven at 105 °C to a constant weight. Crude protein was measured via the Kjeldahl method using a Kjeltec System (Foss Tecator AB, Hoganas, Sweden). Crude lipid was assayed with the Soxtec extraction method using Soxtec Avanti 2050 (Foss Tecator AB). Ash was measured with a muffle furnace at 550 °C for 8 h. Before the proximate composition determination, the fish samples were autoclaved at 121 °C for 20 min, followed by homogenization, and then dried at 65 °C for 24 h.

### 2.5. Serum Components Analysis

The contents of total cholesterol (TC), triglycerides (TG), high-density lipoprotein cholesterol (HDL-C), and low-density lipoprotein cholesterol (LDL-C), as well as the activities of alanine aminotransferase (ALT) and aspartate aminotransferase (AST) in the serum, were assayed via the use of their respective kits produced by Jiancheng Bioengineering Institute (Nanjing, China) following the manufacturer’s instructions. The TC and TG content were measured through the COD-PAP method using the multifunctional enzyme marker (Infinite 200pro, Tecan Austria GimbH, Shanghai, China); the HDL-C and LDL-C content were measured via the peroxidase colorimetric method using the multifunctional enzyme marker (Infinite 200pro); ALT and AST were measured via the Reitman-Frankel method using a visible light spectrophotometer (V-1100D, MAPADA, Shanghai, China).

### 2.6. Fatty Acid Analysis

The total lipids of muscle, liver, and diet samples were extracted via homogenization in a chloroform/methanol (2:1, vol/vol) solution according to [44], and were determined gravimetrically after drying a 5 mL aliquot under nitrogen. The freeze-dried lipid samples (~100 mg) were added into a 25 mL volumetric screwed glass tube with a plastic lid, followed by the addition of 3 mL 2% KOH methanol solution, and were then incubated in a water bath at 75 °C for 20 min. After cooling, the 25 mL glass tube was added with 3 mL 14% boron trifluoride methanol solution, mixed thoroughly, and then the procedure was performed with a water bath at 85 °C for another 20 min. After completing the FA methylation treatment, the resultant solution was transferred to a 10 mL centrifuge tube, followed by the addition of 2 mL double distilled water and 2 mL n-hexane in sequence. The mixture was then shaken vigorously for 1 min, and allowed to separate into 2 layers. The upper layer containing FA methyl esters was separated for subsequent GC analysis.

Fatty acids were determined using gas chromatography (Agilent 7890B-GC, Fairborn Precision Instruments Co., Ltd., Shanghai, China) equipped with a flame ionization detector (FID) and a CD-2560 capillary column (100 m × 0.25 mm × 0.2 μm). When performing measurements, 1 μL of the methylated sample was injected in the split mode at a 50:1 ratio. The column carrier gas was nitrogen, applied at a constant flow rate of 1.25 mL/min. The injector temperature and the FID temperature were set at 250 °C and 270 °C, respectively. The oven temperature was programmed from an initial temperature of 50 °C for 2 min, followed by increments of 5 °C/min until reaching a final temperature of 270 °C for 2 min. The FA component was estimated according to the retention time of standard FAs, and the data were collected via peak area normalization with C19 alkanoic acid (Sigma-Aldrich Shanghai Trading Co., Ltd., Shanghai, China) used as an internal standard.

### 2.7. RNA Extraction and Expression Analysis

The total RNA extraction of individual livers was performed using SYBR Premix Ex Taq Kit (Takara, Dalian, China), followed by the quantification for the total RNA concentration and purity via spectrophotometry, and the quality check through the use of agarose gel electrophoresis. The reverse transcription was completed with one microgram of total RNA using a reverse transcription kit (Thermo). The targeted genes were expressed via quantitative real-time PCR (qRT-PCR) under an ABI 7500 real-time PCR Detection system (Applied Biosystems, Foster City, CA, USA) using SYBR Green Real-time PCR Master Mix (Toyobo, Shanghai, China). The primers for the amplification of gene-specific PCR products were designed through the Primer-BLAST (https://www.ncbi.nlm.nih.gov/tools/primer-blast/, accessed on 7 December 2023); the information of primers for qRT-PCR are shown in Table 3. All primers were commercially provided by Integrated DNA Technologies (Hunan Accurate biological engineering Co., Ltd., Changsha, China). The real-time PCR procedure included a pre-denaturation step at 95 °C for 30 s, 40 cycles at 95 °C for 5 s, annealing and extension temperatures at 60 °C for 30 s, and the final dissociation. The final step was performed to ensure a single product was amplified. The qRT-PCR efficiency (E) was achieved with the equation E = 10^(−1/slope)^. Only after the primers were verified with an efficiency of approximately 100% through amplification can the gene expression results be analyzed using the 2^−ΔΔCt^ method [45]; the data for all treatments were compared to the control group. β-actin was used as the internal reference, and its expression maintains relatively stable in the study.

### 2.8. Calculations

Weight gain (WG, %) = 100 × (final body weight (g/fish) − initial body weight (g/fish))/initial body weight (g/fish)

Specific growth rate (SGR, %/d) = 100 × (ln (finial body weight (g/fish)) − ln (initial body weight(g/fish)))/feeding duration (days)

Daily feed intake (DFI, %/d) = 100 × (feed intake (g/fish)/((final body weight (g/fish) + initial body weight (g/fish))/2 × days))

Feed efficiency (FE) = 100 × ((final body weight (g/fish) − initial body weight (g/fish))/feed intake (g/fish))

Survival (%) = 100 × (final number of fish)/(initial number of fish)

Hepatosomatic index (HSI, %) = 100 × (liver weight (g/fish)/body weight of sample fish (g/fish))

Condition factor (CF, g/cm^3^) = 100 × (body weight of sample fish (g/fish)/(body length (cm/fish))^3^)

Intraperitoneal fat rate (IPF, %) = 100 × (intraperitoneal fat weight (g/fish)/body weight of sample fish (g/fish)).

### 2.9. Statistical Analysis

The data were subjected to analysis of variance (ANOVA) to determine if significant differences occurred among treatments of CO replacing FO after the confirmation of the normality and homogeneity of variance through the Kolmogorov–Smirnov test and Levene’s test in SPSS Statistics 22.0 (SPSS, Michigan Avenue, Chicago, IL, USA). Data presented in percentages or ratios were subjected to data transformation before statistical analysis. The significance of linear or quadratic models were analyzed using orthogonal polynomial contrasts in order to describe the response of the dependent variable to CO inclusion levels. The results were given as the mean and standard deviation (SD). *p* values < 0.05 were considered statistically significant.

## 3. Results

### 3.1. Growth Performance

The results of the growth performance are presented in Table 4. The WG, SGR, FE, and survival were significantly affected when replacing FO with CO in feed (*p* < 0.05). The WG, SGR, FE, and HSI showed positive linear and/or quadratic responses to increasingly replacing FO with CO in diets, peaking at 25% CO for the four parameters. The value for the HSI followed the same trend, reaching its maximum at 100% CO. There were no differences in the DFI and CF among the dietary treatments. According to the quadratic regression analysis of WG against the level of replacing FO with CO, the dietary optimal CO level for the WG of groupers was estimated to be 29.39% of feed (y = −0.0174x^2^ + 1.0226x + 278.96, R^2^ = 0.869, *p* = 0.012).

### 3.2. Proximate Composition, Liver Lipid Content, and Intraperitoneal Fat Rate

As shown in Table 5, the proximate composition of the whole body and muscle was not affected by the dietary treatments (*p* > 0.05). However, a negative quadratic response was observed for the whole-body protein and muscle moisture with increasing levels of CO replacement, with the minimum occurring for the 50% CO diet; the opposite trend was observed for muscle protein and lipid contents, with the maximum being observed for the 50% CO diet. The IPF value and liver lipid content had positive linear and quadratic responses (*p* < 0.05) with increasing levels of FO being replaced with CO, with a peak at 100% CO both for the IPF and liver lipid.

### 3.3. Serum Components

Serum components are shown in Table 6. The serum contents of TC and HDL-C and of ALT activity showed linear and quadratic responses (*p* < 0.05) to increasing dietary carnosine levels, with a maximum for TC at 25% CO, and for HDL-C and ALT at 100% CO; however, the serum contents of TG and LDL-C and of AST activity were not influenced by the dietary treatments (*p* > 0.05).

### 3.4. Tissue Fatty Acid Profile

As shown in Table 7 and Table 8, all the FA contents, except for liver C16:0, showed either positive or negative linear and quadratic responses (*p* < 0.05) both in the liver and in the muscle, with increasing dietary CO inclusion levels. The n-3/n-6PUFA ratios had negative linear and quadratic responses (*p* < 0.05) both in the liver and in the muscle, with the lowest (*p* < 0.05) value found for the 100% CO diet. However, the DHA/EPA ratio in the liver was not affected by the dietary treatments; whereas the values in the muscle exhibited negative linear and quadratic responses (*p* < 0.05), with increasing dietary CO inclusion levels, with the lowest (*p* < 0.05) value found for the 100% CO diet. The FA composition in the liver and muscle almost reflected the FA composition in the feed.

### 3.5. Gene Expression Related to Lipid Metabolism in the Liver

Table 9 shows the relative mRNA expressions of genes related to lipid metabolism in the liver of orange-spotted groupers. The mRNA levels of *fas*, *acc*, *g6pd*, *srebp-1c*, and *δ6fad* generally displayed positive linear and/or quadratic responses (*p* < 0.05) with increasing CO inclusion levels, reaching the highest value for the 100% CO diet for these genes. On the contrary, the values for *elovl 4* and *elovl 5* generally exhibited negative linear and/or quadratic responses (*p* < 0.05) with increasing CO inclusion levels, reaching the lowest value for the 100% CO diet for the two genes. When compared with the control diet, 25% and 50% CO diets up-regulated (*p* < 0.05) the mRNA levels of *cpt 1*, while 75% and 100% CO diets down-regulated (*p* < 0.05) its mRNA levels. The mRNA levels of *hsl* and *atgl* were down-regulated (*p* < 0.05) by dietary CO addition. The mRNA levels of *lpl* were unaffected (*p* > 0.05) by the dietary treatments.

## 4. Discussion

The results of our study indicate that groupers fed diets with up to 100% CO had a comparable growth performance with those being fed the control diet, which was consistent with the observations that mostly or completely replacing FO with CO did not affect the growth performance in other studies with fish [30,32,33,46,47]. In this study, the DHA and EPA contents in the 100% CO diet came entirely from a 30% fish meal containing 6% FO in this feed. This means that 1.8% FO in a 100% CO diet may meet low levels of the EFA requirements of groupers [48]. The findings suggest that diets with high or 100% CO replacement can provide a low level of the EFA demand (such as DHA and EPA) for the growth of juvenile groupers under the current feeding regime, which further explained why groupers achieved a reasonable growth performance when given high CO feed. Recent studies have showed that the inclusion level of EPA + DHA to support the optimal health of fish, such as Atlantic salmon, is much higher than the level of the EFA requirement [49]. From the perspective of the entire production cycle, further verification is needed to determine whether the low level of EPA + DHA can keep the normal growth and health levels of fish at the finishing stage. When EFAs meet the growth requirements, it is natural for VOs containing a large amount of non-EFAs to replace FO in a large proportion [1,50]. The findings are in accordance with the results of previous studies on other fish that were fed high CO diets [30,33]. Moreover, the percent of WG showed a quadratic response and peaked with the 25% CO diet, being significantly higher than that of the control diet, as observed with a peak for the 50% CO replacement diets in previous studies in large yellow croaker [11] and Nile tilapia [34] species, indicating a synergistic effect on growth. In other studies with golden pompano [12] and gibel carp [51], 100% CO diets also exhibited superior growth performance when compared to the FO diet. The enhanced feed utilization and/or feed intake may contribute to growth promotion, as observed in our current study and a previous study [34], a result of the enhanced protein and energy metabolism of fish that consume a certain amount of SFAs [52,53] due to the preferential utilization of SFAs and MUFAs over PUFAs in fish [15,54,55]. However, there are some studies reporting a decrease in fish growth when fish were fed diets with high CO [35,37,38,56,57].

It is clear that MCFAs, rather than LC-PUFAs, are preferentially utilized as substrates for FA β-oxidation to provide more fuel for fish [58]. However, higher levels of MCFAs did not further promote the β-oxidation of FAs for energy supply [19]. Therefore, liver and intraperitoneal lipid accumulation occurred and exhibited an increasing trend with increasing levels of CO replacing FO in this study. This dose-dependent relationship has also been observed in previous studies with other fish species [11,12], indicating that dietary high CO inclusion may be the cause of increased lipid deposition in the liver and other visceral adipose tissues. This may partly explain why suitable levels of SFAs in feed promotes fish growth, while excessive SFAs intake causes inferior growth and/or an increase in lipid deposition in the body [59,60], as evidenced by an elevation in the HSI or/and lipid contents of the whole body and liver, as well as the IPF in this study and previous studies when fish were fed high CO diets [11,12,34].

The results of our current study showed that the proximate compositions of both the whole body and muscle were not influenced by CO replacing FO, which was supported by what has been reported in previous studies [11,32,34,47,51,61]. Inconsistent with the aforementioned, the whole-body lipid content was elevated [12,21,33], but was reduced [37] in CO inclusion diets vs. FO diets. The inconsistency may be attributed to differences in ingredients, fish species, dietary lipid levels, and FA profiles.

Blood biochemical components are metabolic intermediates of nutrients and are usually used to measure the nutritional status of fish [62]. In this study, we observed that both the serum LDL-C and TC had linear and quadratic responses, reaching a peak at the 25% CO inclusion level, while the serum HDL-C followed the similar trend with a peak being found at the 100% CO inclusion level, with increasing levels of CO replacing FO. The aforementioned changes were paralleled with the growth of orange-spotted groupers, reflecting two different states of lipid metabolism of fish fed diets with optimal CO levels and high CO levels. Inconsistent with our observations, the plasma TC was not affected by CO replacing FO in Nile tilapia [21,33] and gibel carp [51]. We also observed an increasing trend of serum ALT activity with increasing levels of CO replacing FO, which disagreed with studies with golden pompano [12] and Nile tilapia [34], whose ALT values were not affected when replacing FO with CO. The elevated serum ALT activity may be attributed to high CO inclusion in higher dietary lipid levels in our current study (12% feed lipid) when compared to the latter (7% and 5.8% feed lipid for golden pompano and Nile tilapia, respectively). This means that the restricted growth performance of orange-spotted groupers was associated with the enhanced serum ALT activity, accompanied by a significant increase in liver lipid deposition caused by dietary 75% and 100% CO inclusion, suggesting the reflection of liver lipid metabolic disturbance [63], potentially as a result of some lipid imbalance between lipid synthesis, β-oxidation, and transport in the liver [49,64,65].

It is clear that the FA compositions in the liver and fillet of fish are influenced by the FA composition in the feed in previous studies [66,67,68,69,70,71,72] and studies from our research team [10,14]. Interestingly, the n-3/n-6 PUFA ratios in the liver and dorsal muscle were positively correlated with those in the feeds and displayed a declining trend with response to the incremental CO level in the feed, reflecting the FA profile of the diet, as evidenced by our previous studies [10,14]. As mentioned above, a lower n-3/n-6 PUFA ratio in the feed promotes the transportation of MCFAs, linoleic acid, and linolenic acid into intermediate metabolism for energy production, but triggers DHA deposition in the liver and muscle [15,55,58,73]. Therefore, it is understandable that the lower n-3/n-6 PUFA ratio in the diets with above 75% CO enhanced a significant lipid accumulation in the liver [11,32], accompanied with the increased enrichment of SFAs and the decreased enrichment of LC-PUFAs in our current research and previous research on PO substitution for FO [14]. Thus, it can be seen that a high CO inclusion level in feeds will compromise the nutritional value of fish flesh. In addition, there were identical DHA/EPA ratios in the tested feeds, and the liver DHA/EPA ratios did not alter across the dietary treatments, but the DHA/EPA ratios in the muscle showed a decreasing trend with increasing CO levels in feed. This indicates that the DHA and EPA contents in the muscle are more susceptible to the influence of dietary CO levels than in the liver [66], reflecting the different utilization and deposition of DHA and EPA by fish [15].

The homeostasis of lipid metabolism in cells of vertebrates mainly involves two important processes: lipid synthesis and decomposition [64]. The key enzymes that regulate FA synthesis include fas, acc, and g6pd, while the key enzymes involved in lipid degradation include lpl, atgl, and hsl [64,65]. The cpt 1 transports LC-PUFAs into mitochondria for β-oxidation [52]. The induction of lipolytic gene expression activates cpt 1 activity, thereby promoting FA β-oxidation [74,75]. The *srebp* regulates metabolic enzyme genes including the *acc*, *fas*, and *scd* involved in FA biosynthesis in vertebrate cells [76]. On the contrary, the *pparα* activation by n-3 LC-PUFA induces lipolytic gene expression and subsequently increases cpt-1 activity, thus enhancing FA β-oxidation [74,75]. In this study, with high levels of FO being replaced with CO, the significant elevation of the SFA content was at the expense of the significant decline of the LC-PUFA content, accompanied with up-regulating the expression of *fas*, *acc*, *g6pd*, and *srebp-1c* genes. Similarly, the enhanced expression of *fas*, *acc*, and/or *g6pd* genes occurred for fish consuming a diet with high or complete levels of CO replacing FO [11,12,32,33,37]. It is clear that high levels of n-3 LC-PUFAs (DHA or/and EPA) down-regulate *fas*, *acc*, and/or *srebp-1c* in gilthead sea bream [77], Atlantic salmon [78], black seabream [37,79], and rainbow trout [80], while low n-3 LC-PUFA levels up-regulate the expression of FA synthesis genes in black seabream [79] as a result of inhibitory FA synthesis genes originating from PUFAs rather than SFAs [81]. Therefore, an enhanced lipogenesis is induced via the up-regulation of the abovementioned genes due to the decrease in the dietary n-3 LC-PUFA levels [79]. On the other hand, although the mRNA level of *pparα* was not determined in this study, the expression level of the *cpt 1* gene was up-regulated with the 25–50% CO levels and down-regulated with the 75–100% CO levels. Similarly, a down-regulated expression of *pparα* and *cpt-1* was observed in large yellow croakers fed a 100% CO substitution diet [37], indicating decreased lipolysis. These findings show a declined β-oxidation, resulting in a reduction in liver lipid consumption in fish fed higher CO diets, eventually promoting an enhanced lipid accumulation in the hepatocytes [82]. However, the expression of the *lpl* gene remained unchanged, which means that the gene does not influence FA uptake by tissues [65]. Meanwhile, the expression levels of the *atgl* and *hsl* genes were down-regulated with the high levels of CO replacing FO in this study and in previous studies [37], but the opposite results occurred in large yellow croakers [11] and largemouth bass [33] when fed high CO diets. The discrepancy may be attributed to the varied degrees of β-oxidation in tissues due to the different FA compositions [79].

The delta-6 fatty acyl desaturases (δfads) and elongases of very long-chain fatty acids (elovls) mediate the LC-PUFA synthesis [83,84]. The former converts LA and LNA into LC-PUFA, and the latter acts as the first step of the FA elongation process [85,86]. In the present study, we observed an up-regulated expression of the *δ6fad* gene and the down-regulated expression of the *elovl 4* and *elovl 5* genes in orange-spotted groupers who were fed high CO diets, which was in accordance with our recently published results concerning FO being replaced with soybean oil or PO in the same fish species [10,14], as well as previous studies where CO replaced FO in black seabream [37]. However, the up-regulated expression of *elovl 5* genes was observed in black seabream when 100% of the FO was replaced with soybean oil or PO [37], Atlantic cod when 100% of the FO was replaced with camelina oil [87,88], and Atlantic salmon when FO was replaced with PO [66]. The conflicting results indicate that the fish species and VO source may determine the extent to which different fish species biosynthesize LC-PUFAs from LA and LNA. In addition, the expression levels of the *δ6fad* gene were positively correlated with, and the expression levels of the *elovl 4* and *elovl 5* genes were negatively correlated with the levels of CO replacing FO in this study, but the LC-PUFA contents were not promoted significantly, reflecting the weak ability of marine or carnivorous fish to synthesize LC-PUFAs.

## 5. Conclusions

This study showed that the growth rate, feed utilization, HSI, IPF, liver lipid content, serum HDL-C content, and serum ALT activity had positive linear and/or quadratic responses, but the serum TC and LDL-C contents exhibited the opposite trend with increasing levels of CO replacing FO. Feeding a 25% CO feed caused the highest growth and feed utilization, due to the synergistic effect of blending FO and CO at a suitable ratio. The FA profile in the liver and muscle of orange-spotted groupers generally mirrored the FA profile in the feed. The significantly elevated liver lipid accumulation caused by high CO diets may be attributed to the lack of further increases in the SFAs used for energy consumption and the alterations of gene expression related to FA metabolism. However, the gene expression changes related to FA metabolism did not promote the n-3 LC-PUFA content of the liver and muscle, though an enhanced biotransformation from 18C FAs to LC-PUFAs was observed when fish were fed a high CO diet with a n-3 LC-PUFA deficiency.

## Figures and Tables

**Table 1:** Ingredients and proximate composition of experimental diets (on an as-fed basis, %).

Item	Diets ^1^				
	0% CO	25% CO	50% CO	75% CO	100% CO
Ingredients					
Fish meal	30	30	30	30	30
Wheat gluten meal	10	10	10	10	10
Soybean meal	28	28	28	28	28
Gelatin (food grade)	1.75	1.75	1.75	1.75	1.75
Casein (food grade)	7	7	7	7	7
Corn starch	11	11	11	11	11
Fish oil	8	6	4	2	0
Coconut oil	0	2	4	6	8
Soy lecithin	2	2	2	2	2
Vitamin premix	0.3	0.3	0.3	0.3	0.3
Mineral premix	0.5	0.5	0.5	0.5	0.5
Stay-C 35%	0.02	0.02	0.02	0.02	0.02
Mold inhibitors Inhibitor	0.1	0.1	0.1	0.1	0.1
Feed antioxidants	0.03	0.03	0.03	0.03	0.03
Ca(H_2_PO_4_)_2_	1.5	1.5	1.5	1.5	1.5
Choline chloride	0.3	0.3	0.3	0.3	0.3
Proximate composition					
Dry matter	96.33	96.06	96.37	95.63	95.52
Crude protein	50.46	50.06	50.94	50.23	50.22
Crude lipid	12.22	12.50	12.65	12.68	12.72
Ash	8.54	8.51	8.48	8.54	8.30

FO, fish oil; CO, coconut oil; 0% CO, basal diet without CO addition. ^1^ Coconut oil replaced 0%, 25%, 50%, 75%, and 100% of FO in the basal diet, respectively. All the ingredients were obtained from Jiakang Feed Co., Ltd., Xiamen, China. The vitamin and trace mineral contents are provided in the vitamin and trace mineral premix according to the study of [14]. Mold inhibitors: 50% calcium propionic acid and 50% dimethyl fumarate. Feed antioxidants: 50% ethoxyquin and 50% butylated hydroxytoluene.

**Table 2:** Fatty acid profiles of the experimental diets (mg/g lipid).

Fatty Acids	FO	CO	Diets ^1^				
			0% CO	25% CO	50% CO	75% CO	100% CO
C10:0	ND	49.2	ND	12.3	19.0	24.9	33.3
C12:0	0.72	408.8	6.4	70.9	114.0	189.2	253.6
C14:0	49.4	151.6	53.8	83.0	110.9	137.6	176.8
C16:0	140.1	60.4	171.9	161.8	156.0	150.9	143.8
C18:0	29.6	21.7	49.5	41.7	33.5	30.4	21.9
ΣSFA	219.8	691.9	281.6	369.8	433.5	533.0	629.6
C16:1n-7	73.0	0.1	37.4	32.9	23.5	13.2	9.1
C18:1n-9 (OA)	179.6	29.6	98.3	92.7	90.4	64.6	44.3
ΣMUFA	252.6	29.7	135.7	125.6	114.0	77.8	53.4
C18:2n-6 (LA)	62.4	4.4	87.1	73.8	56.4	42.9	32.7
C20:3n-6	6.5	ND	21.9	9.7	5.8	2.1	0.8
C20:4n-6 (ARA)	8.1	ND	2.3	1.3	0.9	0.7	ND
Σn-6PUFA	77.1	4.4	111.4	84.8	63.2	45.7	33.5
C18:3n-3 (LNA)	20.5	ND	18.7	11.1	7.6	1.0	ND
C20:5n-3 (EPA)	66.2	ND	42.1	33.3	24.8	17.6	11.7
C22:6n-3 (DHA)	88.3	ND	64.3	47.5	34.2	24.9	16.5
Σn-3PUFA	175.1	ND	125.1	91.8	66.6	43.5	28.2
DHA/EPA	1.33	-	1.53	1.43	1.38	1.42	1.41
n-3/n-6PUFA	2.27	-	1.12	1.08	1.06	0.95	0.85

FO, fish oil; CO, coconut oil; 0% CO, basal diet without CO addition. ^1^ Coconut oil replaced 0%, 25%, 50%, 75%, and 100% of FO in the basal diet, respectively. SFA, saturated fatty acids; MUFA, monounsaturated fatty acids; PUFA, polyunsaturated fatty acid; LC-PUFA, long-chain polyunsaturated fatty acids; OA, oleic acid; LA, linoleic acid; ARA, arachidonic acid; LNA, linolenic acid; DHA, docosahexaenoic acid; EPA, eicosapentaenoic acid; ND, not detected.

**Table 3:** Primer sequences used for real-time PCR.

Genes	Forward Primer Sequence (5′-3′)	Reverse Primer Sequence (5′-3′)	E-Value (%)	KEGG No.
*β-Actin*	TGCTGTCCCTGTATGCCTCT	CCTTGATGTCACGCACGAT	104.0	AY510710
*fas*	GGCAAGCCACTCTGGTACAT	GGCTATGTCTGACCGCAGAA	97.4	FJ196231
*acc*	GGTGGGATACCTACTGGGGT	GGGAACCATACCTGTCCTGC	110.1	FJ196229
*lpl*	CATCGTGATACACGGCTGGA	CAATCACATTGGCACTGGGC	103.3	EU683732
*δ6fad*	CTCATCATTTGGGTCTGGG	GAAGATGTTGGGTTTAGCG	102.0	EU715405
*hsl*	CAGTTAAGGTGAACCGGGCT	ATCTGAACTGGAGCAGTGCC	114.6	KF049203
*atgl*	TGACAACCTGCCTCAGTACG	TGGATGCTCGTGTTGGTGAA	102.8	KY649281
*g6pd*	GGCGAACCGTCTCTTCTACC	CCTGTTCCAGCCTTTTGTGC	111.4	XM010731710
*srebp-1c*	GGTTCAAACCATGGCACCAC	GTCGTGCTTCAGAGTGGTCA	103.8	KT937284
*cpt 1*	AGGGCCGTTTCTACAAGGTG	GCGGCTAGTTTCTCCTCTCC	107.5	HM037343
*elovl 4*	CTTTCATCATCCTCTTCGCC	TTACTCCCTTTTCGCTCGTC	96.0	KF533722
*elovl 5*	GCCTGTGCCAGACAAGGTTA	GCGTCCGGACAATAACCAGA	98.9	KU179484

*fas*, fatty acid synthase; *acc*, acetyl-CoA carboxylase; *lpl*, lipoprotein lipase; *δ6fad*, delta-6 fatty acyl desaturase; *hsl*, hormone-sensitive lipase; *atgl*, adipose triglyceride lipase; *g6pd*, glucose-6-phosphate dehydrogenase; *srebp-1c*, sterol-regulatory element-binding protein-1c; *cpt*, carnitine palmitoyltransferase; *elovl*, elongase of very long-chain fatty acids.

**Table 4:** Effects of fish oil being replaced with coconut oil in feed on the growth performance of orange-spotted grouper fish.

Items	Diets ^1^					Linear		Quadratic	
	0% CO	25% CO	50% CO	75% CO	100% CO	*p*-Value	R^2^	*p*-Value	R^2^
IBW (g)	22.34 ± 0.02	22.35 ± 0.03	22.34 ± 0.04	22.38 ± 0.03	22.34 ± 0.01				
FBW (g)	84.08 ± 4.47 ^ab^	91.21 ± 5.09 ^b^	88.60 ± 3.31 ^b^	77.74 ± 2.37 ^ab^	72.49 ± 1.11 ^a^				
WG (%)	271.0 ± 16.1 ^ab^	308.5 ± 22.3 ^b^	289.7 ± 23.2 ^ab^	238.4 ± 14.8 ^ab^	215.8 ± 10.4 ^a^	0.017	0.418	0.012	0.587
SGR (%/d)	2.36 ± 0.09 ^ab^	2.51 ± 0.10 ^b^	2.46 ± 0.07 ^b^	2.22 ± 0.06 ^ab^	2.10 ± 0.03 ^a^	0.019	0.405	0.007	0.630
FE (%)	114.3 ± 4.8 ^b^	116.5 ± 2.6 ^b^	115.1 ± 3.2 ^b^	98.6 ± 3.6 ^a^	92.8 ± 4.0 ^a^	0.001	0.622	0.001	0.725
DFI (%/d)	1.80 ± 0.20	1.90 ± 0.05	1.81 ± 0.02	1.80 ± 0.00	1.79 ± 0.12	0.950	<0.001	0.602	0.119
HSI (%)	2.26 ± 0.04 ^ab^	2.17 ± 0.01 ^a^	2.34 ± 0.09 ^ab^	2.41 ± 0.04 ^ab^	2.69 ± 0.15 ^b^	0.010	0.463	0.040	0.474
CF (g/cm^3^)	2.96 ± 0.07	2.98 ± 0.04	3.00 ± 0.02	2.83 ± 0.06	2.89 ± 0.02	0.067	0.252	0.193	0.259
Survival (%)	98.67 ± 1.33	100.00 ± 0.00	96.00 ± 2.31	96.00 ± 0.00	94.00 ± 1.33	0.010	0.433	0.044	0.434

IBW, initial body weight; FBW, final body weight; WG, weight gain; SGR, specific growth rate; FE, feed efficiency; DFI, daily feed intake; HSI, hepatosomatic index. FO, fish oil; CO, coconut oil; 0% CO, basal diet without CO addition. ^1^ Coconut oil replaced 0%, 25%, 50%, 75%, and 100% of FO in the basal diet, respectively. Values in the same row with different superscripts indicated significant difference (*p* < 0.05).

**Table 5:** Effects of replacing fish oil with coconut oil on the tissue composition of orange-spotted groupers (%).

Items	Diets ^1^					Linear		Quadratic	
	0% CO	25% CO	50% CO	75% CO	100% CO	*p*-Value	R^2^	*p*-Value	R^2^
Whole body									
Moisture	66.44 ± 1.83	66.02 ± 0.90	68.68 ± 1.14	67.09 ± 1.84	69.67 ± 0.37	0.105	0.221	0.100	0.368
Crude protein	19.40 ± 0.19	18.86 ± 0.21	18.53 ± 0.16	18.67 ± 0.19	18.68 ± 0.06	0.139	0.573	0.048	0.952
Crude lipid	7.60 ± 0.16	7.78 ± 0.15	7.50 ± 0.30	7.41 ± 0.00	6.90 ± 0.25	0.071	0.716	0.052	0.948
Ash	5.12 ± 0.29	5.28 ± 0.25	4.76 ± 0.16	4.80 ± 0.06	4.54 ± 0.05	0.063	0.279	0.078	0.399
Muscle									
Moisture	76.38 ± 0.22	75.59 ± 0.30	75.09 ± 0.20	75.37 ± 0.45	75.98 ± 0.12	0.603	0.101	0.012	0.988
Crude protein	20.14 ± 0.11	20.56 ± 0.19	20.88 ± 0.03	20.85 ± 0.34	20.52 ± 0.09	0.306	0.333	0.015	0.985
Crude lipid	3.02 ± 0.09	3.28 ± 0.30	3.49 ± 0.00	3.03 ± 0.20	2.54 ± 0.02	0.349	0.290	0.045	0.955
Ash	1.28 ± 0.05	1.33 ± 0.02	1.26 ± 0.03	1.31 ± 0.04	1.33 ± 0.05	0.310	0.079	0.383	0.148
IPF	1.34 ± 0.12 ^a^	1.20 ± 0.09 ^a^	1.41 ± 0.13 ^a^	1.70 ± 0.07 ^ab^	1.99 ± 0.17 ^b^	0.002	0.246	0.002	0.324
Liver lipid	19.08 ± 1.04 ^a^	19.29 ± 0.09 ^a^	21.15 ± 0.10 ^ab^	23.36 ± 0.34 ^b^	26.77 ± 1.12 ^c^	0.001	0.792	<0.001	0.894

FO, fish oil; CO, coconut oil; 0% CO, basal diet without CO addition; IPF, Intraperitoneal fat rate. ^1^ Coconut oil replaced 0%, 25%, 50%, 75%, and 100% of FO in the basal diet, respectively. Values in the same row with different superscripts indicated significant difference (*p* < 0.05).

**Table 6:** Effects of replacing fish oil with coconut oil on the serum components of orange-spotted groupers.

Items	Diets ^1^					Linear		Quadratic	
	0% CO	25% CO	50% CO	75% CO	100% CO	*p*-Value	R^2^	*p*-Value	R^2^
TG (mmol/L)	1.23 ± 0.15	1.47 ± 0.02	1.08 ± 0.06	1.04 ± 0.03	1.20 ± 0.16	0.436	0.211	0.766	0.234
TC (mmol/L)	2.13 ± 0.10 ^c^	2.19 ± 0.18 ^c^	1.80 ± 0.10 ^bc^	1.61 ± 0.07 ^ab^	1.29 ± 0.07 ^a^	<0.001	0.794	0.002	0.802
HDL-C (mmol/L)	2.54 ± 0.00 ^a^	2.63 ± 0.16 ^a^	2.81 ± 0.12 ^a^	2.89 ± 0.01 ^a^	3.24 ± 0.00 ^b^	0.001	0.786	0.002	0.826
LDL-C (mmol/L)	1.14 ± 0.46	1.31 ± 0.01	1.29 ± 0.05	1.01 ± 0.07	0.69 ± 0.03	0.560	0.146	0.013	0.987
AST (U/L)	26.06 ± 0.00	20.64 ± 2.91	21.81 ± 1.37	26.34 ± 4.49	28.74 ± 2.82	0.372	0.268	0.168	0.832
ALT (U/L)	26.15 ± 10.11 ^a^	41.21 ± 5.01 ^ab^	54.37 ± 3.57 ^bc^	72.03 ± 4.18 ^c^	74.63 ± 2.56 ^c^	<0.001	0.878	<0.001	0.914

FO, fish oil; CO, coconut oil; 0% CO, basal diet without CO addition; IPF, Intraperitoneal fat rate. TG, triglyceride; TC, total cholesterol; HDL-C, high-density lipoprotein cholesterol; LDL-C, low-density lipoprotein cholesterol; ALT, alanine aminotransferase; AST, aspartate aminotransferase. ^1^ Coconut oil replaced 0%, 25%, 50%, 75%, and 100% of FO in the basal diet, respectively. Values in the same row with different superscripts indicated significant difference (*p* < 0.05).

**Table 7:** Effects of replacing fish oil with coconut oil on liver fatty acid profiles of orange-spotted groupers (mg/g lipid).

Fatty Acid	Diets ^1^					Linear		Quadratic	
	0% CO	25% CO	50% CO	75% CO	100% CO	*p*-Value	R^2^	*p*-Value	R^2^
C10:0	ND	0.23 ± 0.02 ^a^	0.37 ± 0.02 ^b^	0.65 ± 0.02 ^c^	1.05 ± 0.04 ^d^	<0.001	0.919	<0.001	0.937
C12:0	0.61 ± 0.02 ^a^	8.56 ± 0.08 ^b^	23.99 ± 0.30 ^c^	40.41 ± 3.90 ^d^	52.57 ± 0.48 ^e^	<0.001	0.937	<0.001	0.940
C14:0	22.84 ± 0.21 ^a^	40.67 ± 0.07 ^b^	46.50 ± 0.91 ^c^	66.48 ± 0.06 ^d^	81.27 ± 0.53 ^e^	<0.001	0.943	<0.001	0.945
C16:0	143.89 ± 2.36 ^c^	141.17 ± 1.15 ^bc^	137.71 ± 0.67 ^ab^	135.70 ± 0.47 ^a^	135.07 ± 0.79 ^a^	<0.001	0.731	<0.001	0.776
C18:0	43.97 ± 0.23 ^a^	52.05 ± 0.61 ^b^	57.72 ± 0.22 ^c^	62.89 ± 0.60 ^d^	71.79 ± 1.33 ^e^	<0.001	0.954	<0.001	0.954
ΣSFA	211.31 ± 2.33 ^a^	242.69 ± 1.65 ^b^	266.30 ± 1.92 ^c^	306.13 ± 3.75 ^d^	341.75 ± 1.91 ^e^	<0.001	0.951	<0.001	0.955
C16:1n-7	54.25 ± 0.22 ^e^	53.06 ± 0.25 ^d^	49.18 ± 0.26 ^c^	41.52 ± 0.34 ^b^	39.60 ± 0.45 ^a^	<0.001	0.898	<0.001	0.908
C18:1n-9 (OA)	101.00 ± 0.63 ^c^	98.53 ± 0.22 ^b^	97.98 ± 0.38 ^b^	97.68 ± 0.23 ^b^	95.63 ± 0.42 ^a^	<0.001	0.767	<0.001	0.767
ΣMUFA	155.26 ± 0.85 ^d^	151.60 ± 0.46 ^c^	147.16 ± 0.30 ^b^	139.20 ± 0.11 ^a^	135.23 ± 0.11 ^a^	<0.001	0.932	<0.001	0.939
C18:2n-6 (LA)	86.49 ± 0.86 ^c^	82.84 ± 1.18 ^b^	80.29 ± 0.19 ^a^	79.24 ± 0.59 ^a^	77.71 ± 0.36 ^a^	<0.001	0.761	<0.001	0.796
C20:4n-6 (ARA)	21.33 ± 0.16 ^e^	17.73 ± 0.14 ^d^	11.52 ± 0.32 ^c^	8.72 ± 0.34 ^b^	4.57 ± 0.30 ^a^	<0.001	0.904	<0.001	0.907
C20:3n-6	3.49 ± 0.10 ^c^	3.10 ± 0.08 ^b^	2.91 ± 0.09 ^b^	2.21 ± 0.12 ^a^	1.99 ± 0.09 ^a^	<0.001	0.956	<0.001	0.957
Σn-6PUFA	111.31 ± 0.90 ^e^	103.67 ± 1.07 ^d^	94.71 ± 0.45 ^c^	90.17 ± 0.33 ^b^	84.28 ± 0.34 ^a^	<0.001	0.926	<0.001	0.932
C18:3n-3 (LNA)	12.00 ± 0.09 ^c^	10.99 ± 0.75 ^c^	10.61 ± 0.09 ^b c^	9.46 ± 0.09 ^b^	6.72 ± 0.32 ^a^	<0.001	0.846	<0.001	0.903
C20:5n-3 (EPA)	29.71 ± 0.39 ^e^	24.80 ± 0.13 ^d^	20.29 ± 0.50 ^c^	16.73 ± 0.38 ^b^	12.48 ± 0.27 ^a^	<0.001	0.957	<0.001	0.958
C22:6n-3 (DHA)	40.77 ± 0.23 ^e^	34.03 ± 0.65 ^d^	26.23 ± 0.74 ^c^	22.99 ± 0.54 ^b^	16.18 ± 0.31 ^a^	<0.001	0.943	<0.001	0.946
Σn-3PUFA	82.48 ± 0.56 ^e^	69.82 ± 1.25 ^d^	57.12 ± 0.58 ^c^	49.18 ± 0.72 ^b^	35.39 ± 0.86 ^a^	<0.001	0.961	<0.001	0.961
DHA/EPA	1.37 ± 0.01	1.37 ± 0.03	1.29 ± 0.06	1.38 ± 0.04	1.30 ± 0.01	0.302	0.082	0.569	0.090
n-3/n-6PUFA	0.74 ± 0.01 ^d^	0.67 ± 0.02 ^d^	0.61 ± 0.00 ^c^	0.55 ± 0.01 ^b^	0.42 ± 0.01 ^a^	<0.001	0.943	<0.001	0.958

FO, fish oil; CO, coconut oil; 0% CO, basal diet without CO addition; SFA, saturated fatty acids; MUFA, monounsaturated fatty acids; PUFA, polyunsaturated fatty acid; OA, oleic acid; LA, linoleic acid; ARA, arachidonic acid; LNA, linolenic acid; DHA, docosahexaenoic acid; EPA, eicosapentaenoic acid; ND, not detected. ^1^ Coconut oil replaced 0%, 25%, 50%, 75%, and 100% of FO in the basal diet, respectively. Values in the same row with different superscripts indicated significant difference (*p* < 0.05).

**Table 8:** Effects of replacing fish oil with coconut oil on muscle fatty acid profiles of orange-spotted groupers (mg/g lipid).

Fatty Acid	Diets ^1^					Linear		Quadratic	
	0% CO	25% CO	50% CO	75% CO	100% CO	*p*-Value	R^2^	*p*-Value	R^2^
C10:0	ND	3.13 ± 0.14 ^a^	5.33 ± 0.08 ^b^	9.05 ± 0.37 ^c^	10.14 ± 0.04 ^d^	<0.001	0.946	<0.001	0.951
C12:0	3.13 ± 0.15 ^a^	42.33 ± 0.57 ^b^	91.37 ± 0.32 ^c^	136.11 ± 0.51 ^d^	167.29 ± 1.88 ^e^	<0.001	0.960	<0.001	0.962
C14:0	42.40 ± 0.55 ^a^	56.19 ± 0.42 ^b^	68.55 ± 2.89 ^c^	92.43 ± 1.44 ^d^	108.93 ± 0.75 ^e^	<0.001	0.953	<0.001	0.960
C16:0	142.68 ± 1.70	145.45 ± 5.09	145.88 ± 0.60	145.20 ± 0.68	145.05 ± 1.13	0.634	0.018	0.788	0.039
C18:0	54.33 ± 0.50 ^e^	51.13 ± 0.45 ^d^	45.25 ± 0.65 ^c^	42.81 ± 0.42 ^b^	38.39 ± 0.19 ^a^	<0.001	0.932	<0.001	0.933
ΣSFA	242.55 ± 1.03 ^a^	298.24 ± 4.99 ^b^	356.36 ± 3.51 ^c^	425.60 ± 2.20 ^d^	469.80 ± 2.28 ^e^	<0.001	0.959	<0.001	0.959
C16:1n-7	51.49 ± 1.71 ^e^	47.84 ± 0.82 ^d^	36.23 ± 0.23 ^c^	24.80 ± 0.14 ^b^	19.15 ± 0.97 ^a^	<0.001	0.931	<0.001	0.934
C18:1n-9 (OA)	102.20 ± 0.72	94.65 ± 3.86	93.92 ± 0.08	92.75 ± 3.86	92.85 ± 0.28	0.020	0.351	0.039	0.417
ΣMUFA	153.68 ± 1.00 ^d^	142.49 ± 3.80 ^c^	130.16 ± 0.15 ^b^	117.55 ± 3.81 ^a^	111.99 ± 0.82 ^a^	<0.001	0.915	<0.001	0.917
C18:2n-6 (LA)	89.23 ± 0.00 ^d^	79.09 ± 0.09 ^c^	75.65 ± 0.25 ^b^	72.89 ± 2.26 ^a^	71.01 ± 0.16 ^a^	<0.001	0.809	<0.001	0.890
C20:4n-6 (ARA)	13.78 ± 0.29 ^e^	11.74 ± 0.59 ^d^	7.57 ± 0.38 ^c^	4.99 ± 0.26 ^b^	1.89 ± 0.10 ^a^	<0.001	0.929	<0.001	0.930
C20:3n-6	13.13 ± 0.10 ^e^	11.51 ± 0.13 ^d^	7.28 ± 0.37 ^c^	4.89 ± 0.10 ^b^	3.00 ± 0.02 ^a^	<0.001	0.940	<0.001	0.941
Σn-6PUFA	116.14 ± 0.31 ^e^	102.35 ± 0.68 ^d^	90.50 ± 0.36 ^c^	82.77 ± 2.46 ^b^	75.89 ± 0.05 ^a^	<0.001	0.927	<0.001	0.943
C18:3n-3 (LNA)	13.25 ± 0.22 ^e^	10.38 ± 0.20 ^d^	8.58 ± 0.13 ^c^	6.43 ± 0.09 ^b^	5.90 ± 0.03 ^a^	<0.001	0.929	<0.001	0.966
C20:5n-3 (EPA)	38.58 ± 0.18 ^e^	33.85 ± 0.25 ^d^	27.38 ± 0.62 ^c^	22.43 ± 0.09 ^b^	19.00 ± 0.21 ^a^	<0.001	0.952	<0.001	0.956
C22:6n-3 (DHA)	59.68 ± 0.63 ^e^	51.92 ± 0.70 ^d^	40.98 ± 1.15 ^c^	33.19 ± 0.04 ^b^	26.53 ± 0.28 ^a^	<0.001	0.953	<0.001	0.955
Σn-3PUFA	111.51 ± 0.31 ^e^	96.15 ± 0.98 ^d^	76.93 ± 1.75 ^c^	62.04 ± 0.15 ^b^	51.44 ± 0.47 ^a^	<0.001	0.954	<0.001	0.959
DHA/EPA	1.55 ± 0.02 ^c^	1.52 ± 0.01 ^c^	1.50 ± 0.01 ^bc^	1.48 ± 0.01 ^b^	1.40 ± 0.00 ^a^	<0.001	0.765	<0.001	0.840
n-3/n-6PUFA	0.96 ± 0.00 ^d^	0.94 ± 0.02 ^d^	0.85 ± 0.02 ^c^	0.75 ± 0.02 ^b^	0.68 ± 0.01 ^a^	<0.001	0.904	<0.001	0.912

FO, fish oil; CO, coconut oil; 0% CO, basal diet without CO addition; SFA, saturated fatty acids; MUFA, monounsaturated fatty acids; PUFA, polyunsaturated fatty acid; OA, oleic acid; LA, linoleic acid; ARA, arachidonic acid; LNA, linolenic acid; DHA, docosahexaenoic acid; EPA, eicosapentaenoic acid; ND, not detected. ^1^ Coconut oil replaced 0%, 25%, 50%, 75%, and 100% of FO in the basal diet, respectively. Values in the same row with different superscripts indicated significant difference (*p* < 0.05).

**Table 9:** Effects of replacing fish oil with coconut oil on the relative expression of genes related to fat metabolism in the liver of orange-spotted groupers.

Gene	Diets ^1^					Linear		Quadratic	
	0% CO	25% CO	50% CO	75% CO	100% CO	*p*-Value	R^2^	*p*-Value	R^2^
*fas*	1.00 ± 0.02 ^a^	1.16 ± 0.00 ^a^	1.50 ± 0.01 ^a^	1.67 ± 0.01 ^a^	2.30 ± 0.22 ^b^	0.007	0.938	0.021	0.919
*acc*	1.00 ± 0.00 ^a^	1.46 ± 0.00 ^b^	1.19 ± 0.04 ^ab^	1.44 ± 0.17 ^b^	1.95 ± 0.07 ^c^	0.081	0.831	0.255	0.745
*g6pd*	1.00 ± 0.02 ^a^	1.09 ± 0.04 ^a^	1.24 ± 0.10 ^a^	1.42 ± 0.17 ^ab^	1.68 ± 0.09 ^b^	0.003	0.966	<0.001	1.000
*srebp-1c*	1.00 ± 0.01	1.28 ± 0.21	1.35 ± 0.12	1.46 ± 0.09	1.56 ± 0.16	0.008	0.973	0.029	0.971
*cpt 1*	1.00 ± 0.02 ^a^	1.34 ± 0.17 ^b^	2.20 ± 0.06 ^c^	0.87 ± 0.01 ^a^	0.72 ± 0.12 ^a^	0.653	0.076	0.410	0.590
*hsl*	1.00 ± 0.02 ^b^	0.68 ± 0.09 ^a^	0.72 ± 0.08 ^a^	0.59 ± 0.03 ^a^	0.62 ± 0.05 ^a^	0.088	0.676	0.136	0.864
*atgl*	1.00 ± 0.01 ^b^	0.35 ± 0.04 ^a^	0.35 ± 0.05 ^a^	0.38 ± 0.01 ^a^	0.32 ± 0.03 ^a^	0.169	0.521	0.172	0.828
*lpl*	1.00 ± 0.08	0.88 ± 0.06	1.26 ± 0.05	1.10 ± 0.14	1.01 ± 0.06	0.662	0.072	0.721	0.279
*δ6fad*	1.00 ± 0.06 ^a^	1.36 ± 0.29 ^b^	2.32 ± 0.11 ^c^	2.54 ± 0.00 ^c^	2.89 ± 0.33 ^c^	0.005	0.949	0.035	0.965
*elovl 4*	1.00 ± 0.09 ^b^	0.89 ± 0.09 ^b^	0.64 ± 0.06 ^a^	0.63 ± 0.03 ^a^	0.43 ± 0.06 ^a^	0.005	0.952	0.047	0.953
*elovl 5*	1.00 ± 0.03 ^b^	0.70 ± 0.13 ^ab^	0.82 ± 0.07 ^ab^	0.60 ± 0.04 ^a^	0.56 ± 0.05 ^a^	0.057	0.753	0.229	0.771

*fas*, fatty acid synthase; acc, acetyl-CoA carboxylase; *lpl*, lipoprotein lipase; *δ6fad*, delta-6 fatty acyl desaturase; *hsl*, hormone-sensitive lipase; *atgl*, adipose triglyceride lipase; *g6pd*, glucose-6-phosphate dehydrogenase; *srebp-1c*, sterol-regulatory element-binding protein-1c; *cpt*, carnitine palmitoyltransferase; *elovl*, elongase of very long-chain fatty acids. ^1^ Coconut oil replaced 0%, 25%, 50%, 75%, and 100% of FO in the basal diet, respectively. Values in the same row with different superscripts indicated significant difference (*p* < 0.05).

## Data Availability

The data that support the findings of this study are available from the corresponding author upon reasonable request.

## References

[1] Turchini G.M., Torstensen B.E., Ng W. (2009). Fish oil replacement in finfish nutrition. Rev. Aquacult..

[2] Nasopoulou C., Zabetakis I. (2012). Benefits of fish oil replacement by plant originated oils in compounded fish feeds. A review. Food Sci. Technol..

[3] Fukada H., Kitagima R., Shinagawa J., Morino H., Masumoto T. (2019). Effects of complete replacement of fish oil with plant oil mixtures and algal meal on growth performance and fatty acid composition in juvenile yellowtail *Seriola quinqueradiata*. Fish. Sci..

[4] Tocher D.R., Betancor M.B., Sprague M., Olsen R.E., Napier J.A. (2019). Omega-3 long-chain polyunsaturated fatty acids, EPA and DHA: Bridging the gap between supply and demand. Nutrients.

[5] Benedito-Palos L., Navarro J.C., Sitja-Bobadilla A., Bell J.G., Kaushik S., Perez-Sanchez J. (2008). High levels of vegetable oils in plant protein-rich diets fed to gilthead sea bream (*Sparus aurata* L.): Growth performance, muscle fatty acid profiles and histological alterations of target tissues. Br. J. Nutr..

[6] Abbasi A., Oujifard A., Torfi Mozanzadeh M., Habibi H., Nafisi Bahabadi M. (2020). Dietary simultaneous replacement of fish meal and fish oil with blends of plant proteins and vegetable oils in yellowfin seabream (*Acanthopagrus latus*) fry: Growth, digestive enzymes, antioxidant status and skin mucosal immunity. Aquacult. Nutr..

[7] Alvarez A., Fontanillas R., Hernández-Contreras A., Hernández M.D. (2020). Partial replacement of fish oil with vegetal oils in commercial diets: The effect on the quality of gilthead seabream (*Sparus aurata*). Anim. Feed Sci. Technol..

[8] Chen Y., Sun Z., Liang Z., Xie Y., Su J., Luo Q., Zhu J., Liu Q., Han T., Wang A. (2020). Effects of dietary fish oil replacement by soybean oil and l-carnitine supplementation on growth performance, fatty acid composition, lipid metabolism and liver health of juvenile largemouth bass, *Micropterus salmoides*. Aquaculture.

[9] Fountoulaki E., Vasilaki A., Hurtado R., Grigorakis K., Karacostas I., Nengas I., Rigos G., Kotzamanis Y., Venou B., Alexis M.N. (2009). Fish oil substitution by vegetable oils in commercial diets for gilthead sea bream (*Sparus aurata* L.); effects on growth performance, flesh quality and fillet fatty acid profile recovery of fatty acid profiles by a fish oil finishing diet under fluctuating water temperatures. Aquaculture.

[10] He L., Qin Y., Wang Y., Li D., Chen W., Ye J. (2021). Effects of dietary replacement of fish oil with soybean oil on the growth performance, plasma components, fatty acid composition and lipid metabolism of groupers *Epinephelus coioides*. Aquacult. Nutr..

[11] Ding T., Xu N., Liu Y., Li X., Xiang X., Xu D., Yao C., Liu Q., Yin Z., Mai K. (2020). Optimal amounts of coconut oil in diets improve the growth, antioxidant capacity and lipid metabolism of large yellow croaker (*Larimichthys crocea*). Mar. Life Sci. Technol..

[12] Guo H., Chen C., Yan X., Li Y., Wen X., You C., Monroig Ó., Tocher D.R., Wang S. (2021). Effects of different dietary oil sources on growth performance, antioxidant capacity and lipid deposition of juvenile golden pompano *Trachinotus ovatus*. Aquaculture.

[13] Lim P., Boey P., Ng W. (2001). Dietary palm oil level affects growth performance, protein retention and tissue vitamin E concentration of African catfish, *Clarias gariepinus*. Aquaculture.

[14] Qin Y., He L., Wang Y., Li D., Chen W., Ye J. (2022). Growth performance, fatty acid composition, and lipid metabolism are altered in groupers (*Epinephelus coioides*) by dietary fish oil replacement with palm oil. Anim. Nutr..

[15] Turchini G.M., Francis D.S., Senadheera S.P.S.D., Thanuthong T., De Silva S.S. (2011). Fish oil replacement with different vegetable oils in Murray cod: Evidence of an “omega-3 sparing effect” by other dietary fatty acids. Aquaculture.

[16] Wallace T.C. (2019). Health Effects of coconut oil-A narrative review of current evidence. J. Am. Coll. Nutr..

[17] Marina A.M., Che Man Y.B., Nazimah S.A.H., Amin I. (2009). Chemical properties of virgin coconut oil. J. Am. Oil Chem. Soc..

[18] Wang J., Wang X., Li J., Chen Y., Yang W., Zhang L. (2015). Effects of dietary coconut oil as a medium-chain fatty acid source on performance, carcass composition and serum lipids in male broilers. Asian-Austral. J. Anim. Sci..

[19] Smith D.M., Williams I.H., Williams K.C., Barclay M.C., Venables W.N. (2015). Oxidation of medium-chain and long-chain fatty acids by polka dot grouper Cromileptes altivelis. Aquacult. Nutr..

[20] Bhatnagar A.S., Kumar P.K.P., Hemavathy J., Krishna A.G.G. (2009). Fatty acid composition, oxidative stability, and radical scavenging activity of vegetable oil blends with coconut oil. J. Am. Oil Chem. Soc..

[21] Apraku A., Liu L., Leng X., Rupia E.J., Ayisi C.L. (2017). Evaluation of blended virgin coconut oil and fish oil on growth performance and resistance to Streptococcus iniae challenge of Nile tilapia (*Oreochromis niloticus*). Egypt. J. Basic Appl. Sci..

[22] Dayrit F.M. (2015). Properties of lauric acid and their significance in coconut oil. J. Am. Oil Chem. Soc..

[23] Ng Y.J., Tham P.E., Khoo K.S., Cheng C.K., Chew K.W., Show P.L. (2021). A comprehensive review on the techniques for coconut oil extraction and its application. Bioprocess. Biosyst. Eng..

[24] Prabha S., Tamoli S., Raghavamenon A.C., Manu K.A. (2024). Virgin coconut oil alleviates dextran sulphate-induced inflammatory bowel disease and modulates inflammation and immune response in mice. J. Am. Nutr. Assoc..

[25] Vysakh A., Ratheesh M., Rajmohanan T.P., Pramod C., Premlal S., Girish K.B., Sibi P.I. (2014). Polyphenolics isolated from virgin coconut oil inhibits adjuvant induced arthritis in rats through antioxidant and anti-inflammatory action. Int. Immunopharmacol..

[26] Sacks F.M. (2020). Coconut oil and heart health: Fact or fiction?. Circulation.

[27] Spiazzi B.F., Duarte A.C., Zingano C.P., Teixeira P.P., Amazarray C.R., Merello E.N., Wayerbacher L.F., Farenzena L.P., Correia P.E., Bertoluci M.C. (2023). Coconut oil: An overview of cardiometabolic effects and the public health burden of misinformation. Arch. Endocrinol. Metab..

[28] Swarnamali H., Ranasinghe P., Jayawardena R. (2024). The effect of coconut oil and palm oil on anthropometric parameters: A clinical trial. BMC Nutr..

[29] Orsavova J., Misurcova L., Ambrozova J.V., Vicha R., Mlcek J. (2015). Fatty acids composition of vegetable oils and its contribution to dietary energy intake and dependence of cardiovascular mortality on dietary intake of fatty acids. Int. J. Mol. Sci..

[30] Babalola T.O., Apata D.F. (2012). Effects of dietary lipid source on growth, digestibility and tissue fatty acid composition of *Heterobranchus longifilis* fingerlings. J. Agric. Rural. Dev. Trop..

[31] Karanth S., Sharma P., Pal A.K., Venkateshwarlu G. (2009). Effect of different vegetable oils on growth and fatty acid profile of Rohu (*Labeo rohita*, Hamilton); Evaluation of a return fish oil diet to restore human cardio-protective fatty acids. Asian-Australas. J. Anim. Sci..

[32] Liang C., Zhao X., Jiao L., Shen Y., Luo J., Zhu T., Zhao W., Gen Z., Zhou Q., Jin M. (2022). Effects of different lipid sources on growth performance, fatty acids composition in tissue and expression of genes related to lipid metabolism in largemouth bass (*Micropterus salmoides*). Aquacult. Rep..

[33] Zhang W., Sun S., Ge X., Zhu J., Miao L., Lin Y., Su Y., Liang H., Pan W., Yu H. (2018). Effects of dietary lipid sources on growth performance, fatty acid composition and hepatic lipid metabolism of juvenile blunt snout bream, *Megalobrama amblycephala*. Aquacult. Nutr..

[34] Dawood M., Ali M.F., Amer A.A., Gewaily M.S., Mahmoud M.M., Alkafafy M., Assar D.H., Soliman A.A., Van Doan H. (2021). The influence of coconut oil on the growth, immune, and antioxidative responses and the intestinal digestive enzymes and histomorphometry features of Nile tilapia (*Oreochromis niloticus*). Fish Physiol. Biochem..

[35] Corrêa C.F., Nobrega R.O., Mattioni B., Block J.M., Fracalossi D.M. (2017). Dietary lipid sources affect the performance of Nile tilapia at optimal and cold, suboptimal temperatures. Aquacult. Nutr..

[36] Abdel-Ghany H.M., El-Sayed A.M., Ezzat A.A., Essa M.A., Helal A.M. (2019). Dietary lipid sources affect cold tolerance of Nile tilapia (*Oreochromis niloticus*). J. Therm. Biol..

[37] Jin M., Yuan Y., Lu Y., Ma H.N., Sun P., Li Y., Qiu H., Ding L.Y., Zhou Q.C. (2017). Regulation of growth, tissue fatty acid composition, biochemical parameters and lipid related genes expression by different dietary lipid sources in juvenile black seabream, *Acanthopagrus schlegelii*. Aquaculture.

[38] Tseng Y., Lin Y.H. (2020). Effects of dietary supplementation with coconut oil on the growth, fatty acid profiles and some lipid metabolism relative gene expressions of orange-spotted grouper *Epinephelus coioides*. Aquacult. Nutr..

[39] Shapawi R., Abdullah F.C., Senoo S., Mustafa S. (2019). Nutrition, growth and resilience of tiger grouper (*Epinephelus fuscoguttatus*) × giant grouper (*Epinephelus lanceolatus*) hybrid—A review. Rev. Aquacult..

[40] China Bureau of Fisheries (2023). China Fishery Statistics Yearbook.

[41] Shapawi R., Mustafa S., Ng W.K. (2008). Effects of dietary fish oil replacement with vegetable oils on growth and tissue fatty acid composition of humpback grouper, *Cromileptes altivelis* (Valenciennes). Aquac. Res..

[42] Gudid S.N., Lim L.S., Yong A.S.K., Yanuhar U., Shapawi R. (2020). Growth performance and organoleptic quality of hybrid grouper (*Epinephelus fuscogutattus* ♀ *× Epinephelus lanceolatus* ♂) fed palm-oil based diets at grow-out stage. Sains Malays..

[43] AOAC (1995). Official Methods of Analysis of AOAC International.

[44] Folch J., Lees M., Sloane S.G. (1957). A simple method for the isolation and purification of total lipides from animal tissues. J. Biol. Chem..

[45] Schmittgen T.D., Livak K.J. (2008). Analyzing real-time PCR data by the comparative C(T) method. Nat. Protoc..

[46] Craig S.R., Gatlin R.D.M. (1995). Coconut oil and beef tallow, but not tricaprylin, can replace menhaden oil in the diet of red drum (*Sciaenops ocellatus*) without adversely affecting growth or fatty acid composition. J. Nutr..

[47] Figueiredo-Silva A.C., Kaushik S., Terrier F., Schrama J.W., Médale F., Geurden I. (2012). Link between lipid metabolism and voluntary food intake in rainbow trout fed coconut oil rich in medium-chain TAG. Brit. J. Nutr..

[48] Wu F.C., Ting Y.Y., Chen H.Y. (2002). Docosahexaenoic acid is superior to eicosapentaenoic acid as the essential fatty acid for growth of grouper, *Epinephelus malabaricus*. J. Nutr..

[49] Tocher D.R., Sprague M., Han L., Sayanova O., Norambuena F., Napier J.A., Betancor M.B. (2024). Inclusion of oil from transgenic Camelina sativa in feed effectively supplies EPA and DHA to Atlantic salmon (*Salmo salar*) grown to market size in seawater pens. Food Chem..

[50] Bordignon F., Tomas-Vidal A., Trocino A., Milian S.M., Jover-Cerda M., Martinez-Llorens S. (2020). Fatty acid signatures in different tissues of mediterranean yellowtail, *Seriola dumerili* (Risso, 1810), fed diets containing different levels of vegetable and fish oils. Animals.

[51] Chen J.L., Han D., Zhu X.M., Yang Y.X., Xie S.Q. (2011). Dietary lipid sources for gibel carp *Carassius auratus* gibelio: Growth performance, tissue composition and muscle fatty acid profiles. Acta Hydrobiol. Sin..

[52] Lu K.L., Xu W.N., Wang L.N., Zhang D.D., Zhang C.N., Liu W.B. (2014). Hepatic beta-oxidation and regulation of carnitine palmitoyltransferase (CPT) I in blunt snout bream *Megalobrama amblycephala* fed a high fat diet. PLoS ONE.

[53] National Research Council (NRC) (2011). Nutrient Requirements of Fish and Shrimp.

[54] Henderson R.J. (1996). Fatty acid metabolism in freshwater fish with particular reference to polyunsaturated fatty acids. Arch. Für Tierernährung.

[55] Rombenso A.N., Trushenski J.T., Drawbridge M. (2018). Saturated lipids are more effective than others in juvenile California yellowtail feeds—Understanding and harnessing LC-PUFA sparing for fish oil replacement. Aquaculture.

[56] Davis D., Lazo J., Arnold C. (1999). Response of juvenile red drum (*Sciaenops ocellatus*) to practical diets supplemented with medium chain triglycerides. Fish Physiol. Biochem..

[57] Williams I., Williams K.C., Smith D.M., Jones M. (2006). Polka-dot grouper, *Cromileptes altivelis*, can utilize dietary fat efficiently. Aquacult. Nutr..

[58] Schönfeld P., Wojtczak L. (2016). Short- and medium-chain fatty acids in energy metabolism: The cellular perspective. J. Lipid Res..

[59] Elmsjö A., Rosqvist F., Engskog M.K.R., Haglöf J., Kullberg J., Iggman D., Johansson L., Ahlström H., Arvidsson T., Risérus U. (2015). NMR-based metabolic profiling in healthy individuals overfed different types of fat: Links to changes in liver fat accumulation and lean tissue mass. Nutr. Diabetes.

[60] Huang X., Bian C., Ji H., Ji S., Sun J. (2023). DHA induces adipocyte lipolysis through endoplasmic reticulum stress and the cAMP/PKA signaling pathway in grass carp (*Ctenopharyngodon idella*). Anim. Nutr..

[61] Ballestrazzi R., Rainis S., Maxia M. (2006). The replacement of fish oil with refined coconut oil in the diet of large rainbow trout (*Oncorhynchus mykiss*). Ital. J. Anim. Sci..

[62] Song T., Qin Y., Ke L., Wang X., Wang K., Sun Y., Ye J. (2022). Dietary lactoferrin supplementation improves growth performance and intestinal health of juvenile orange-spotted groupers (*Epinephelus coioides*). Metabolites.

[63] Zhang M., Xue M., Xiao Z., Liu W., Jiang N., Meng Y., Fan Y., Liu X., Zhou Y. (2022). *Staphylococcus sciuri* causes disease and pathological changes in hybrid sturgeon *Acipenser baerii* × *Acipenser schrencki*. Front. Cell. Infect. Microbiol..

[64] Musso G., Gambino R., Cassader M. (2009). Recent insights into hepatic lipid metabolism in non-alcoholic fatty liver disease (NAFLD). Prog. Lipid Res..

[65] Saponaro C., Gaggini M., Carli F., Gastaldelli A. (2015). The subtle balance between lipolysis and lipogenesis: A critical point in metabolic homeostasis. Nutrients.

[66] Bell J.G., Henderson R.J., Tocher D.R., Mcghee F., Dick J.R., Porter A., Smullen R.P., Sargent J.R. (2002). Substituting fish oil with crude palm oil in the diet of Atlantic salmon (*Salmo salar*) affects muscle fatty acid composition and hepatic fatty acid metabolism. J. Nutr..

[67] Deng D.F., Ju Z.Y., Dominy W.G., Conquest L., Smiley S., Bechtel P.J. (2014). Effect of replacing dietary menhaden oil with pollock or soybean oil on muscle fatty acid composition and growth performance of juvenile Pacific threadfin (*Polydactylus sexfilis*). Aquaculture.

[68] Fonseca-Madrigal J., Karalazos V., Campbell P.J., Bell J.G., Tocher D.R. (2005). Influence of dietary palm oil on growth, tissue fatty acid compositions, and fatty acid metabolism in liver and intestine in rainbow trout (*Oncorhynchus mykiss*). Aquacult. Nutr..

[69] Monge Ortiz R., Tomás Vidal A., Rodriguez Barreto D., Martínez Llorens S., Pérez J.A., Jover Cerdá M., Lorenzo A. (2018). Replacement of fish oil with vegetable oil blends in feeds for greater amberjack (*Seriola dumerili*) juveniles: Effect on growth performance, feed efficiency, tissue fatty acid composition and flesh nutritional value. Aquacult. Nutr..

[70] Ng W.K., Tocher D.R., Bell J.G. (2007). The use of palm oil in aquaculture feeds for salmonid species. Eur. J. Lipid Sci. Technol..

[71] Ofori-Mensah S., Yıldız M., Arslan M., Eldem V. (2020). Fish oil replacement with different vegetable oils in gilthead seabream, *Sparus aurata* diets: Effects on fatty acid metabolism based on whole-body fatty acid balance method and genes expression. Aquaculture.

[72] Yu J., Li S., Chang J., Niu H., Hu Z., Han Y. (2019). Effect of variation in the dietary ratio of linseed oil to fish oil on growth, body composition, tissues fatty acid composition, flesh nutritional value and immune indices in Manchurian trout, *Brachymystax lenok*. Aquacult. Nutr..

[73] Bandarra N.M., Rema P., Batista I., Pousão-Ferreira P., Valente L.M.P., Batista S.M.G., Ozório R.O.A. (2011). Effects of dietary n−3/n−6 ratio on lipid metabolism of gilthead seabream (*Sparus aurata*). Eur. J. Lipid Sci. Technol..

[74] Ipsen D.H., Lykkesfeldt J., Tveden-Nyborg P. (2018). Molecular mechanisms of hepatic lipid accumulation in non-alcoholic fatty liver disease. Cell. Mol. Life Sci..

[75] Poudyal H., Panchal S.K., Diwan V., Brown L. (2011). Omega-3 fatty acids and metabolic syndrome: Effects and emerging mechanisms of action. Prog. Lipid Res..

[76] Dong Q., Giorgianni F., Beranova-Giorgianni S., Deng X., O’Meally R.N., Bridges D., Park E.A., Cole R.N., Elam M.B., Raghow R. (2016). Glycogen synthase kinase-3-mediated phosphorylation of serine 73 targets sterol response element binding protein-1c (SREBP-1c) for proteasomal degradation. Biosci. Rep..

[77] Magalhães R., Guerreiro I., Coutinho F., Moutinho S., Sousa S., Delerue-Matos C., Domingues V.F., Olsen R.E., Peres H., Oliva-Teles A. (2020). Effect of dietary ARA/EPA/DHA ratios on growth performance and intermediary metabolism of gilthead sea bream (*Sparus aurata*) juveniles. Aquaculture.

[78] Minghetti M., Leaver M.J., Tocher D.R. (2011). Transcriptional control mechanisms of genes of lipid and fatty acid metabolism in the Atlantic salmon (*Salmo salar* L.) established cell line, SHK-1. Mol. Cell Biol. Lipids.

[79] Jin M., Lu Y., Yuan Y., Li Y., Qiu H., Sun P., Ma H.N., Ding L.Y., Zhou Q.C. (2017). Regulation of growth, antioxidant capacity, fatty acid profiles, hematological characteristics and expression of lipid related genes by different dietary n-3 highly unsaturated fatty acids in juvenile black seabream (*Acanthopagrus schlegelii*). Aquaculture.

[80] Alvarez M.J., Diez A., Lopez-Bote C., Gallego M., Bautista J.M. (2000). Short-term modulation of lipogenesis by macronutrients in rainbow trout (*Oncorhynchus mykiss*) hepatocytes. Br. J. Nutr..

[81] Kralova Lesna I., Suchanek P., Kovar J., Stavek P., Poledne R. (2008). Replacement of dietary saturated FAs by PUFAs in diet and reverse cholesterol transport. J. Lipid Res..

[82] Torstensen B.E., Nanton D.A., Olsvik P.A., Sundvold H., Stubhaug I. (2009). Gene expression of fatty acid binding proteins (FABPs), fatty acid transport proteins (cd36 and FATP) and β-oxidation related genes in Atlantic salmon (*Salmo salar* L.) fed fish oil or vegetable oil. Aquacult. Nutr..

[83] Lee J.H., Giannikopoulos P., Duncan S.A., Wang J., Johansen C.T., Brown J.D., Plutzky J., Hegele R.A., Glimcher L.H., Lee A. (2011). The transcription factor cyclic AMP-responsive element-binding protein H regulates triglyceride metabolism. Nat. Med..

[84] Castro L.F., Tocher D.R., Monroig O. (2016). Long-chain polyunsaturated fatty acid biosynthesis in chordates: Insights into the evolution of Fads and Elovl gene repertoire. Prog. Lipid Res..

[85] Izquierdo M.S., Robaina L., Juarez-Carrillo E., Oliva V., Hernandez-Cruz C.M., Afonso J.M. (2008). Regulation of growth, fatty acid composition and delta 6 desaturase expression by dietary lipids in gilthead seabream larvae (*Sparus aurata*). Fish Physiol. Biochem..

[86] Morais S., Monroig O., Zheng X., Leaver M.J., Tocher D.R. (2009). Highly unsaturated fatty acid synthesis in Atlantic salmon: Characterization of ELOVL5- and ELOVL2-like elongases. Mar. Biotechnol..

[87] Tocher D.R., Zheng X., Schlechtriem C., Hastings N., Dick J.R., Teale A.J. (2006). Highly unsaturated fatty acid synthesis in marine fish: Cloning, functional characterization, and nutritional regulation of fatty acyl delta 6 desaturase of Atlantic cod (*Gadus morhua* L.). Lipids.

[88] Xue X., Feng C.Y., Hixson S.M., Johnstone K., Anderson D.M., Parrish C.C., Rise M.L. (2014). Characterization of the fatty acyl elongase (elovl) gene family, and hepatic elovl and delta-6 fatty acyl desaturase transcript expression and fatty acid responses to diets containing camelina oil in Atlantic cod (*Gadus morhua*). Comp. Biochem. Physiol. B Biochem. Mol. Biol..

